# Toward a Neural Chronometry for the Aesthetic Experience of Music

**DOI:** 10.3389/fpsyg.2013.00206

**Published:** 2013-05-01

**Authors:** Elvira Brattico, Brigitte Bogert, Thomas Jacobsen

**Affiliations:** ^1^Cognitive Brain Research Unit, Institute of Behavioural Sciences, University of HelsinkiHelsinki, Finland; ^2^Finnish Center of Excellence in Interdisciplinary Music Research, University of JyväskyläJyväskylä, Finland; ^3^Brain and Mind Laboratory, Department of Biomedical Engineering and Computational Science, Aalto University School of ScienceHelsinki, Finland; ^4^Experimental Psychology Unit, Faculty of Humanities and Social Sciences, Helmut Schmidt University/University of the Federal Armed Forces HamburgHamburg, Germany

**Keywords:** music emotion, music cognition, liking, preference, appraisal, judgment, aesthetics, brain

## Abstract

Music is often studied as a cognitive domain alongside language. The emotional aspects of music have also been shown to be important, but views on their nature diverge. For instance, the specific emotions that music induces and how they relate to emotional expression are still under debate. Here we propose a mental and neural chronometry of the aesthetic experience of music initiated and mediated by external and internal contexts such as intentionality, background mood, attention, and expertise. The initial stages necessary for an aesthetic experience of music are feature analysis, integration across modalities, and cognitive processing on the basis of long-term knowledge. These stages are common to individuals belonging to the same musical culture. The initial emotional reactions to music include the startle reflex, core “liking,” and arousal. Subsequently, discrete emotions are perceived and induced. Presumably somatomotor processes synchronizing the body with the music also come into play here. The subsequent stages, in which cognitive, affective, and decisional processes intermingle, require controlled cross-modal neural processes to result in aesthetic emotions, aesthetic judgments, and conscious liking. These latter aesthetic stages often require attention, intentionality, and expertise for their full actualization.

## Introduction

An aesthetic experience of music is an important phenomenon worthy of scientific study as testified by questionnaire surveys pointing to enjoyment, beauty, and nostalgia as some of the foremost aesthetic reasons for listening to music (along with entertainment, company and the like; e.g., Laukka, 2007). Even the decision to play an instrument or to choose music as a profession often derives from aesthetic past experiences (Sloboda, 1992; Juslin and Laukka, 2004). In spite of its importance as well as its long history of philosophical and scientific investigation (for a review, see Brattico and Pearce, 2013), aesthetic experience is also one of the most poorly defined concepts in psychology and neuroscience (Juslin et al., 2010; Markovic, 2012). In this paper, utilizing neuroscientific evidence as a starting point, we aim at providing an explicit definition of this phenomenon and its components organized in temporal order. In parallel, we discuss the questions yet unsolved or left open by the available evidence and suggest hypotheses for further testing.

Until recent years, investigations of music within the field of cognitive neuroscience have focused on instrumental music particularly from the Western classical repertoire. The proportion of studies focusing on classical instrumental music, though, is quickly decreasing as in the past few years researchers have been increasingly exploring brain responses to other musical genres as well (e.g., Limb and Braun, 2008; Janata, 2009; Berns et al., 2010; Brattico et al., 2011; Johnson et al., 2011; Montag et al., 2011; Pereira et al., 2011; Salimpoor et al., 2011; Zuckerman et al., 2012; Alluri et al., in press). Cognitive neuroscientists have typically considered music as a perceptual and cognitive phenomenon to be compared to language, memory, attention, and other human cognitive functions (e.g., Peretz and Zatorre, 2003; Koelsch and Siebel, 2005). Until now, the neurosciences of music have given very little attention to the aesthetic aspects of the musical phenomenon, like judgments of the value of music as a form of art (cf. Brattico and Pearce, 2013). Recent attention, however, has been devoted to musical emotions though with clearly divergent views on the kinds of emotions that music truly generates and how these emotions are evoked (see, e.g., Juslin and Västfjäll, 2008; Konecni, 2008; Zentner et al., 2008). Here, we provide a novel perspective of the musical phenomenon as an instance of the aesthetic experience triggered by an object or an external event without an intrinsic survival function. In doing so, we integrate cognitive and affective and decision-making processes related to music in a single mental act, namely the aesthetic experience. Furthermore, following the concept of mental chronometry in associating cognitive operations to brain events happening in real time (Donders, 1869; Posner, 2005), we propose that this aesthetic musical experience consists of a cascade of mental processes in an individual (alone or together with others) occurring at a precise moment in time. We consider not only classical instrumental music consumed in a concert hall but also other very common aesthetic phenomena centered around music, such as ushering at a live pop/rock concert or watching opera. Our descriptions mainly concern the aesthetic experiences of Western individuals as little is known about the content and conceptualization of those experiences in isolated non-Western cultures. We also provide working hypotheses that could help solve outstanding issues on the nature of musical emotions and of the musical phenomenon in its multimodal complexity.

According to Chatterjee’s (2011) fresh conceptualization, aesthetics includes “the perception, production, and response to art, as well as interactions with objects and scenes that evoke an intense feeling, often of pleasure.” Markovic (2012) instead specifically proposes a definition of an aesthetic experience as an “exceptional state of mind,” in which focused attention plays a crucial role, and which responds not to bodily needs (such as appetitive and mating functions) but provides “pleasures for the mind” (p. 2). In our recent review on neuroesthetic studies of music (Brattico and Pearce, 2013), we define an aesthetic experience of music “as one in which the individual immerses herself in the music, dedicating her attention to perceptual, cognitive and affective interpretation based on the formal properties of the perceptual experience.” Aesthetic processing, namely information processing of an artistic object (see, e.g., Jacobsen, 2006), comprises receptive (sensory), central (emotional, perceptual, and cognitive), and productive processes. Aesthetic experience, as defined here and elsewhere (e.g., Leder et al., 2004; Shelley, 2012), comprises only receptive and central processes, resulting in emotions, appreciation, and judgment of a sensorial entity, such as a musical piece, with respect to one or more relevant concepts (like beauty, elegance, rhythm, mastering of performance, and so on). Production is not in the focus of this framework although it might constitute a minor aspect (humming along with a song, tapping with a foot, etc.).

**Figure 1:**
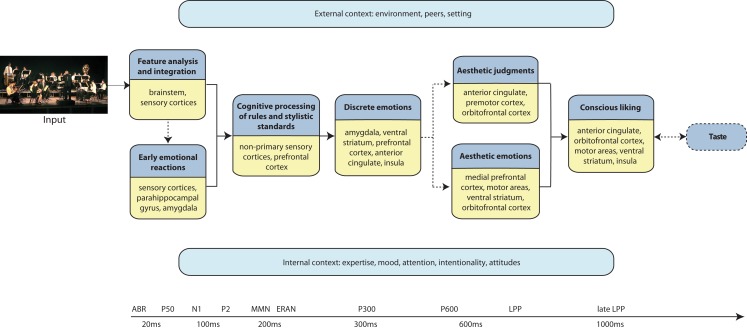
**Schematic representation of the model of the aesthetic experience of music**. Each darker blue box illustrates a processing component, with the brain structures indicated in the accompanying yellow box. The chronological succession of processing flows from left to right. The arrows represent pathways of information flow between processing components. The dashed lines indicate processes that may not always occur during an aesthetic experience of music. The light blue boxes represent the modulatory factors that might affect any of the processing stages of the aesthetic experience of music, according to a different timescale and in a possibly non-linear fashion. A timeline along the bottom roughly indicates the temporal succession of events and the corresponding electrophysiological responses that have been associated to an aesthetic musical experience.

The components of a given aesthetic process are governed by situational and individual characteristics. They also change over time because they are affected by biological and cultural evolution, by technical possibilities and by fashion (together referred to as diachronia). Within a given time, cultural, subcultural, and group factors (together referred to as ipsichronia), determine aesthetic processing as well as specifics of a given content domain (Jacobsen et al., 2004; Jacobsen, 2006, 2010; Brattico et al., 2009; Istók et al., 2009). Here we propose a novel approach in which the components of an aesthetic experience are not static but dynamic modules. The model conforms to an information processing view in which mental events are defined both spatially, namely associated with specific neural locations or networks, and temporally, evolving in time as the outcome of distinct neural mechanisms. (We refer the reader to Figure 1 as a guide to the temporal order of events in the aesthetic experience and the related brain structures). We propose that complete actualization of a musical aesthetic experience requires a particular (aesthetic) attitude, intentionality, attention, and the appropriate context. When those are present, the aesthetic experience comes to full fruition by inducing emotions in the individual (particularly aesthetic ones, defined below; for emotion concepts used in this framework, please see Table 1), by prompting an evaluative judgment of, e.g., beauty, and by determining liking and a time-lasting preference. Hence, by identifying putative temporally and/or spatially quantifiable subprocesses with the help of evidence obtained with functional magnetic resonance imaging (fMRI), positron emission tomography (PET), magnetoencephalography (MEG), and electroencephalography (EEG) as well as from brain-lesioned patients, we provide a workable definition of the musical aesthetic experience for future investigations. Particularly, in our proposal we define three main outcomes of the musical aesthetic experience: aesthetic emotions (e.g., enjoyment, interest, nostalgia), aesthetic judgments (namely, the appraisal of the beauty of a musical piece or the evaluation of the perfection and efficacy of a musical performance), and preference (for instance, the liking or disliking of a piece or a musical genre). All these outcomes of a musical aesthetic experience require perceptual, cognitive, and early emotional reactions to music to come into existence. However, of the three, only aesthetic emotions consist mainly of affective processes, whereas aesthetic judgments and preference include also evaluative, cognitive, and decisional processes. In the following sections, we elaborate on the subprocesses that constitute a musical aesthetic experience along with the underlying neural mechanisms and proposed temporal succession.

**Table 1:** **Glossary of emotion terms and concepts used in the framework**.

Circumplex model of affect	An approach proposing that all affective states arise from two fundamental neurophysiological systems, one related to valence (a pleasure – displeasure continuum) and the other to arousal, or alertness
Valence	A bipolar continuum of stimulus characteristics or emotional experience ranging from negative to neutral to positive
Arousal	A bipolar continuum that varies from calm to excitement
Appraisal theory	A theory proposing that emotions are elicited and differentiated on the basis of a person’s subjective evaluation or appraisal of the personal significance of a situation, object, or event based on a number of dimensions or criteria
Chills	Tremor or tingling sensations passing through the body as the result of sudden keen emotion or excitement
Conceptual-act model	A model of emotions in which the labeling and categorization of core affect states using conceptual knowledge of emotions. This model highlights the fact that affect is a continuum, even though emotions are thought to be discrete
Basic emotion theory	The theory posits that emotions can be divided into discrete and independent categories and that specific neural structures and pathways subserve each of these emotional categories

Our chronometric framework is inspired by important contributions from the field of empirical aesthetics of visual arts: it creatively fuses the previous proposals of an information processing framework including several temporal stages leading to the aesthetic experience of contemporary abstract figurative art by Leder et al. (2004) with a modular framework based on findings from neurological patients by Chatterjee (2003) (see also the complementary proposal by Nadal et al., 2008). Specifically, here we incorporate psychological findings related to musical aesthetic subprocesses with neurophysiological evidence pointing to the temporal order of neural events and with neuroimaging findings identifying the neural structures responsible for those events. Notably, our proposal contains feedback and feedforward loops affecting the processing stages (similar loops have been proposed also in models drawing from music psychology evidence; for a review, see Hargreaves and North, 2010). These stages are hard to draw in a linear fashion as they operate on different time scales, hence it is important to keep in mind that the processes we are going to describe are far more complex than illustrated here. In our framework, we include, for example, expectations derived from implicit memory for a musical style, current mood, and personal strategies of regulating mood by using music, peer influences on a musical aesthetic experience (e.g., how listening to a rap song will affect the impression of peers), intentionality, and so on. It is also important to remind the reader that music is viewed here not only as an “abstract pattern of sound” but also as a multi-systemic communal phenomenon and as an embodied experience, similar to what has been conceptualized by Dissanayake (2008). Therefore while we will concentrate on auditory events in the model due to space limitations and to the paucity of related empirical findings, other motor, visual, and somatosensory aspects of music, when conceived as a performing art rather than as a perceptual and cognitive domain, should be kept in mind and will need to be taken into consideration in the future.

## Stages of Processing in Temporal Order

### Feature analysis and integration

A necessary prerequisite for any aesthetic experience of music is the reception of the musical signal itself. Although a deaf person might have an embodied aesthetic experience by perceiving sound waves as vibrations via the somatosensory receptors in the body, the aesthetic experience does not reach completeness and might lack the full induction of emotions or the formation of a preference for a certain musical genre (even when the hearing deficit is corrected by a cochlear implant, e.g., Hopyan et al., 2012). Music is a highly complex sensory signal that is analyzed in the central nervous system beginning with the peripheral organs and their connections to the sensory cortex (Koelsch, 2011). Because lyrics are present in most music (Nettle, 1983) and often motivate listeners to approach music (Laukka, 2007), the feature analysis relevant to an aesthetic musical experience might encompass not only the acoustic non-speech signal but also the speech and language signal. Moreover, the visual signal must also be taken into account when considering, for instance, attitudes, episodic memory, and affective forecasting, which are among the main modulating factors in the aesthetic experience of music to be described below. As exemplified by the impulse to raise the volume to feel a favorite piece of music with the whole body, somatosensory input presumably enhances the aesthetic enjoyment of music by performers and listeners alike, especially for the diffuse genres of pop and rock during live performances or dance events. Nevertheless, since the acoustic signal is necessary and sometimes even sufficient (e.g., when listening through headphones to an unfamiliar musical piece with eyes closed) for an aesthetic experience of music, we will focus on it here.

The first *feature analysis* of the acoustic signal occurs in the hair cells situated on the basilar membrane of the inner ear. They operate as a fast Fourier transform to decompose the frequencies of complex tones according to the time code for low-frequency range, the space code for high-frequency range, and the volley principle (combining time and space codes: when the waveform peaks occur too fast for a single neuron to fire at each peak, several neurons fire contemporarily to encode the sound frequency) for medium-frequency range tones (e.g., Moore, 1989). Other sound parameters are decoded in the inner ear and in the brainstem. For instance, information on sound localization is extracted only at the level of the inferior colliculus, which receives neural afferents from the contralateral ear (as also demonstrated with electrophysiological auditory brainstem responses or ABR; see Figure 1; e.g., Pickles, 1988). Twenty milliseconds after sound onset, the acoustic signal reaches the primary auditory cortex situated in the medial part of the transverse temporal gyrus, or Heschl’s gyrus (BA 41), in the supratemporal lobes of both cerebral hemispheres. At this point, the sound is still below the level of perception and is not yet an integrated, composite sound percept, as shown by electric intracranial stimulation of the primary auditory cortex (performed presurgically in patients) resulting in isolated sinusoidal sounds and single sound features (e.g., Hall et al., 2003). Spatial encoding is reproduced up to the primary auditory cortex; the tuning curves of individual neurons, which indicate the characteristic frequencies to which they best respond, become wider in secondary (BA 42) and almost non-existent in downstream areas of the auditory cortex.

The ventral stream, starting from the anterior part of the superior temporal gyrus (where the non-primary auditory cortex is located) and continuing to the inferior frontal lobe, is responsible for the *integration* and short-term storage of spectrotemporal sound features into single invariant percepts, sometimes possessing an internal hierarchical structure (Rauschecker, 2011). This integration and storage process, indexed electrophysiologically by the mismatch negativity (MMN) auditory cortex response of the event-related potential (ERP; Näätänen and Winkler, 1999), is crucial for perceiving spectrotemporally complex sound features such as contour (the pattern of ups and downs of pitches; Tervaniemi et al., 2001; Trainor et al., 2002a), intervals (the frequency difference between sounds; Brattico et al., 2000), and Gestalt sound patterns (Shinozaki et al., 2000). Indeed, the superior temporal gyrus anterolateral to Heschl’s gyrus seems to be the neural locus where relations between successive sounds are processed (e.g., Patterson et al., 2002; Samson et al., 2010). Stream segregation is also performed at this stage of processing along with the binding of auditory and cross-modal (e.g., visual) features, as demonstrated by the MMN response (e.g., Takegata et al., 2005). All of these auditory processes are automatic. The anterior non-primary auditory cortex even processes sounds according to the properties of the intervals of a musical scale, producing a neural response if sounds slightly deviate from those properties (Brattico et al., 2001, 2006; Leino et al., 2007). Alternatively, the posterior supratemporal lobe, part of the dorsal stream of auditory processing, supposedly represents a “do pathway” to process and select those complex sounds that will be transformed into representations in the premotor and motor regions (Patterson et al., 2002; Zatorre et al., 2007). The neural responses to musical sounds are mainly generated in the superior temporal gyrus of the right hemisphere, whereas comparable responses to the speech sounds (like phonemes) of a native language predominantly originate in the corresponding area of the left hemisphere (Shestakova et al., 2002).

Rhythm and its perceptual attribute, i.e., the beat (defined as a series of psychological events equally spaced in time; e.g., Grahn, 2009), is an indispensable part of musical engagement and hence its aesthetic experience. When sounds are temporally structured, it is common to nod our heads or tap our feet to the beat. In turn, meter is the pattern of accented (strong and weak) beats unfolding as equal units of time with specific relations to each other (e.g., Zatorre et al., 2007; for musical terms discussed in this paper, please see Table 2). Beat extraction and motor synchronization are fast and automatic processes most likely initiated by oscillators at the level of the medial geniculate nucleus of the thalamus and subsequently (with a lag of about 100 ms) in the auditory cortex, as shown by the corresponding neural responses to phase or tempo perturbations of periodic sequences (Vuust et al., 2011; for a review see Large and Snyder, 2009). Motor areas, including the premotor cortex, the dorsolateral prefrontal cortex, the putamen, and the cerebellum, are also involved in rhythm perception (Chen et al., 2008; Grahn and Rowe, 2009; Alluri et al., 2012) as well as in its production (Chen et al., 2008).

**Table 2:** **Glossary of music terms used in the framework**.

Consonance	The pleasant, “stable” sound sensation produced by certain combinations of two tones played simultaneously
Dissonance	The unpleasant grating sound heard with other sound combinations
Harmony	The use of different pitches simultaneously, namely chords, and the relative conventions related to their succession and voice leading
Meter	The measurement of the number of beats between more or less regularly recurring accents
Timbre	The quality of sound according to which a listener can judge that two sounds similarly presented and with same loudness and pitch are dissimilar. Timbre distinguishes one music instrument from another
Tonality	The organization of pitches in such a way that one central pitch dominates and attracts the others and gives name to the key

### Cognitive processing of rules and stylistic standards

An important step for reaching aesthetic responses to music relies on the implicit or explicit understanding of its formal structure. The importance of this stage of processing for a musical aesthetic experience is exemplified with the neurological disorder of congenital amusia. In amusics, the learning of musical conventions, like harmonic structures, is not possible due to reduced connectivity between frontotemporal brain structures (Peretz et al., 2005; Loui et al., 2009; Hyde et al., 2011). As a result, a music-specific disorder originates that is associated with the active avoidance of aesthetic musical experiences (McDonald and Stewart, 2008). When examining the formal structure of a musical piece, one has to take into account that music, like language, is a complex signal with elements organized according to a culturally determined hierarchy of importance. This includes elements such as: (1) tonality, which establishes the rules for sounds to be included in a tonal composition, (2) harmony, which determines the conventions governing sound succession, and (3) meter, which determines expectations for temporal regularities. (However, the exact nature of metrical hierarchy is still under debate, with traditional theories posing top-down rules and novel ones proposing dynamic attending as a flexible, bottom-up, interaction between external input and internal attending oscillatory processes; Large and Jones, 1999.) Processing these and other hierarchical rules requires the use of higher cognitive functions, including memory and attention, that follow the neural processing of basic sound features but, in our model, precede the conscious perception and induction of emotions.

The detection of an unexpected sound violating the conventions of tonal harmony is dependent on attentional and right-hemispheric processes that occur early in the processing of a musical stimulus. For instance, the discrimination of chord deviations from the conventions of Western tonal harmony requires the integration of auditory events over time by using working memory processes, the hierarchical organization of those events based on schematic knowledge stored in long-term memory, and hence the recruitment of attentional resources and prefrontal brain structures (Koelsch, 2009; Garza Villarreal et al., 2011). Indeed, the electrophysiological response (measured with both EEG and MEG) that has been associated with the *cognitive processing of harmony rules* in the brain is the early right anterior negativity or ERAN, mainly generated by neuronal populations in the inferior frontal gyrus, particularly in Broca’s area (BA 45) and its right analog (Tillmann et al., 2003; Koelsch et al., 2005; Garza Villarreal et al., 2011). Broca’s area is a multimodal brain region that is generally important for hierarchical processing and sequence learning, both in natural and artificial language syntax and in motor domains (Koechlin and Jubault, 2006). The hierarchical processing of rules and conventions of sound successions play a central role in the building of an aesthetic emotional response. Meyer (1956) first theorized that the sources of emotional aesthetic responses to music would include both the expectations based on harmony and tonality rules as well as the tension accumulated as a consequence of their violations and delayed fulfillment. This claim recently found empirical support by means of ERP measures, behavioral ratings, and skin conductance responses (Steinbeis et al., 2006), and has been re-proposed also in the context of the chill response by Vuust and Kringelbach (2010) (see next section).

Another memory system involved in the cognitive processing of music is semantic memory, which is related to long-term concepts and structures such as those characterizing a particular musical style. When listening to a piece of music, familiarity with its musical style determines the formation of online schematic expectations. The anterior temporal cortex of the left hemisphere appears to be particularly involved in semantic memory retrieval for music regardless of contextual information (Platel et al., 2003). Specifically, PET experiment using O^15^ as a measure of metabolic activity, a task in which subjects had to classify whether a flute melody was familiar or unfamiliar to them recruited the bilateral middle temporal gyrus (BA 21), the left inferior frontal gyrus (BA 47), and the left angular gyrus, consistent with what has been found with semantic memory tasks in verbal and visual domains. Although the time course of musical semantic memory is not yet fully understood, presumably, as in speech, the processing of semantic features occurs early in the aesthetic experience.

The perception of tonality, or the hierarchical organization of pitches centered around a single pitch called the “tonic,” largely involves frontal regions of the brain (e.g., Janata, 2005; Alluri et al., 2012). Melodies modulated from one tonality to another activate the medial prefrontal cortex as revealed by fMRI (BA 8/10; Janata et al., 2002, Janata, 2009). This vast area of the frontal lobe is related to a wide range of higher cognitive functions, including self-monitoring, short-term working memory, and self-reflection of one’s own emotional states (Gilbert et al., 2006); thus, it is hardly specific to tonality processing. Janata (2009) also found that activations of the medial prefrontal cortex correlated both with high ratings of memory associations and with the computational tonality tracking of the same songs, and hence suggested a function for the medial prefrontal cortex in binding together music and autobiographical memories to issue the affective reaction of nostalgia. This study exemplifies the non-linear succession of aesthetic processing stages, wherein perceptual processes (extracting the pitches of a song) intermingle and overlap temporally with cognitive (implicit tracking of tonality changes) and emotional (nostalgia) ones. Interestingly, by using a novel method of voxel-based analysis on continuous fMRI data acquired while subjects listened to a whole musical piece, Alluri et al. (2012) replicated and extended the finding by Janata (2009). They confirmed that the presence of an unclear key (extracted computationally) in an 8-min orchestral piece by Piazzolla (“Adios Nonino”) correlated with increases in the fMRI signal in the superior frontal gyrus (BA 9; close to the activation observed by Janata, 2009) and with activity in the primary and supplementary motor areas (BA 3 and 6), the insula, and the rolandic operculum.

To summarize, the frontotemporal networks (corresponding to a ventral or anterior stream of auditory processing; Rauschecker, 2011) of individuals exposed to the same musical culture are responsible for perceptual and cognitive processing of the musical regularities and their violations (with variations according to the levels of familiarity with and expertise in music; to be discussed later), which is a preliminary stage toward perception and induction of emotions (see Figure 1).

### Early emotional reactions: Startle reflex, core liking, and arousal

The first emotional responses to any sound, including those heard within a musical aesthetic context, occur very quickly and in a reflex-like manner. What we call an *early emotional reaction* attributes affective coloratura to what is heard most likely in parallel to feature analysis and the pre-attentive formation of a neural representation for sounds. Juslin and Västfjäll (2008) have distinguished six psychological mechanisms by which music arouses (fast or slow) emotional reactions, namely brainstem reflexes, evaluative conditioning, emotional contagion, visual imagery, episodic memory, and expectancy. In their proposal, though, they did not consider resolving those mechanisms in the temporal dimension. Vuust and Kringelbach (2010) reduce these emotion-inducing mechanisms into three: hardwired responses (corresponding to the brainstem reflexes), extramusical associations (which include evaluative conditioning, emotional contagion, visual imagery, and episodic memory), and anticipation or expectancy. The authors consider the last mechanism, dependent on previous knowledge of music, as the most important one for a hedonic musical experience.

Among the early emotional reactions, brainstem reflexes are fast and automatic, enabling an immediate response to potentially dangerous stimuli, and as such may have had an evolutionary advantage (Juslin and Västfjäll, 2008). The most obvious example of an immediate affective reaction to sounds is the auditory startle reflex (Davis et al., 1982). It occurs automatically as a fast, defensive motor response to surprising sounds, and it is commonly measured as the amplitude of the eye blink to a loud white noise. The neural mechanisms involved include nuclei in the brainstem and possibly the amygdala (for a review, see Peretz, 2010). Interestingly, Roy et al. (2009) found that the startle reflex is attenuated by pleasant consonant sounds, suggesting commonalities between these two affective reactions, i.e., the startle reflex response and the response to pleasant consonant sounds.

Indeed, the second example of early emotional reactions consists of sensory pleasantness or “core liking,” defined by neurobiologists as the physiologically pleasurable experience derived from a stimulus of which the subject is unaware but which subliminally may determine actions and judgments, as demonstrated by priming paradigms (Berridge and Kringelbach, 2008). The circumplex model, a widely adopted account of emotions, also identifies the dimension of valence (along with arousal, to be discussed below) as the positive or negative pleasurable connotations of stimuli (Wundt, 1897, 1998; Russell, 1980). Valence is often studied with introspective methods by asking subjects to rate on discrete scales the pleasantness or unpleasantness of stimuli, in which simple acoustic features have been manipulated. Hence when measuring valence, some mechanisms related to core liking are often also addressed. The positive pole of core liking, exemplified by the sensation of consonance, is possibly an active process (Braun, 1999; Tramo et al., 2003), in which pleasure centers in the brainstem are speedily reached without the mediation of higher-order brain structures. The opposite negative pole, sensory dissonance, lies in the annoying, irritating sensation caused by two simultaneous sounds provoking the firing of hair cells in the basilar membrane less than two thirds of the critical bandwidth apart (Kameoka and Kuriyagawa, 1969; Fishman et al., 2001; Juslin and Västfjäll, 2008; Juslin et al., 2010; Peretz, 2010). Sensory dissonance or “core disliking” is a universal prerequisite of musical skills: it has been identified in many world cultures and in infants as young as 2 months (Trainor et al., 2002b). Furthermore, in monkeys, epileptic patients, and healthy adults, enhanced phase-locked neuronal firing or stimulus-locked brain potentials originating from the primary and non-primary auditory cortices distinguish dissonant from consonant sounds (Brattico et al., 2000, 2009; Fishman et al., 2001; Schön et al., 2005). However, in adult non-musicians, auditory cortex (ERP) responses to dissonant chords occur earlier when averaged according to subjects’ pleasantness ratings than when averaged according to music theory classification of the chords (Schön et al., 2005). These results well illustrate the complex interaction, typical of aesthetic phenomena, between feature analysis, early emotional reactions, and subjective experience (the latter being affected by one’s listening biography and other individual factors). The affective aspects of sensory dissonance are likely encoded in the parahippocampal gyrus as shown by neuroimaging experiments (Blood et al., 1999; Koelsch et al., 2006) and lesion studies (Gosselin et al., 2006).

Another putative aspect of early emotional reactions to music is arousal. According to Berlyne (1971), the hedonic qualities of stimuli, artistic or not, can be traced to their arousal potential, namely their ability to affect a degree of arousal in the autonomic nervous system of the subject (with moderately complex music inducing an optimal level of moderate arousal vs. too complex or too simple music inducing too high or too low arousal, respectively). Stimuli with high arousal power are characterized by novelty, loudness, fast temporal dynamics, or what Berlyne (1971) termed “collative” variables. This model has been heavily criticized in the context of empirical aesthetics of visual art on the basis of the wide variability of the physiological responses to arousing stimuli (cf. Silvia, 2005). Nevertheless, arousal or intensity of energy is still conceptualized and largely used in music emotion literature, e.g., as one dimension of the circumplex model of the emotions expressed and induced by music (e.g., Eerola and Vuoskoski, 2011), the other dimension being valence (Russell, 1980). Both high-arousal music (happy and fearful) compared with relaxing (sad or calm) music and incongruous, unexpected chords compared with expected, congruous chords induce electrodermal changes indicative of sympathetic activity in the autonomous nervous system associated with arousing stimuli (Khalfa et al., 2002; Steinbeis et al., 2006). According to Hargreaves and North (2010), the arousal level of the autonomic nervous system predicts the conscious liking of music and some fine-grained emotional responses related to the listener’s engagement with the music, such as feeling excited, bored, or unsettled. The bilateral superior temporal gyrus, the caudate nucleus within the basal ganglia, the cerebellum, and the motor cortex have been associated with music ratings of high arousal in a recent study by Trost et al. (2012). This fMRI study, however, does not provide any notion of the temporal dynamics of the arousal response. Studies using ERP or other methods with fine temporal resolution should test the hypothesis that the arousal response occurs in an early and automatic way.

According to some authors, arousal and valence (or sensory consonance/dissonance) are central components of an aesthetic experience (for a review of findings and theories in the experimental aesthetics of music, see Hargreaves and North, 2010). Here, however, we consider these as only subprocesses of the musical aesthetic experience, similar to how Lindquist et al. (2012) considers them as the two dimensions of “core affect,” needing conceptual-act and categorization by frontal structures to issue a conscious emotion. In our proposal, arousal and valence are early affective reactions to music, followed by their conscious categorical attribution as discrete emotions, and finally leading to the outcomes of aesthetic emotions, aesthetic judgments, and attitudes.

### Discrete emotions in music

As a combination of several mechanisms (discussed in detail by Juslin et al., 2010), including early emotional reactions to sounds, resemblance to vocalizations or animal calls, imitation, episodic memory, and the fulfillment or violation of culture-based expectancy for incoming sounds, *discrete emotions* are perceived in or even induced by music. Within our proposal, it is crucial to distinguish the concepts of the expression of emotion by music (from the sender), the perception of emotion (by the receiver/listener), and the subjective experience of emotion induced by the music (mostly also for the receiver/listener; Juslin and Laukka, 2004). Although each of those concepts cannot often exist without the others, the separation between them should guide the researcher to pose scientific questions accurately. For instance, the dissociation between the expression of sad, negative emotions by music and the positive, joyful feeling derived by listening to that same music is a common illustration of the intertwining components of an affective experience of music.

The analysis of emotion terms used daily in languages around the world has led to the identification of the existence of seven discrete emotions considered the building blocks of all affective experiences: anger, disgust, fear, sadness, joy, shame, and guilt (Ekman, 1992). According to this theory, discrete emotions are “basic” since they are observable in young children and across cultures (Ekman, 1992). Hence they are supposed to result from species evolution and are characterized by unique neural and physiological processes, or “affect programs” (Tomkins, 1962). The most studied of these basic emotions is fear. The amygdala, which stores emotional memories and provides access to them through associative learning (Phelps and LeDoux, 2005), becomes active even when fearful stimuli are presented in the absence of attention (e.g., Öhman et al., 2007). However, in a meta-analysis of the PET and fMRI literature (Phan et al., 2002), the amygdala was found active in only 60% of studies using fearful stimuli. Competing theories, such as the conceptual-act model of emotions, postulate that core affect is a continuum of neurophysiological states of valence and arousal (similarly to the circumplex model by Russell, 1980), which is visible in specific brain activations. The perception and induction of discrete emotions would then emerge from the categorization and labeling of core affect states “in the moment” using conceptual knowledge of emotions (Barrett, 2006). Within the conceptual-act model, the activation of the amygdala is interpreted as related to uncertainty, salience, and high arousal in external stimuli (Lindquist et al., 2012). However, despite diverging interpretations from competing theories, neuroimaging evidence has robustly identified the brain structures governing the perception and induction of emotional experiences, such as the lateral and ventromedial orbitofrontal cortex, the ventral striatum including the nucleus accumbens, the anterior cingulate cortex (particularly its sub/pregenual portions), the insula (particularly its anterior portion, which keeps track of subjective temporal experiences, and visceral interoceptive states, hence supposedly generating the representation of a “sentient self”; Craig, 2010), and other subcortical areas that govern autonomic reactions to emotions, such as the thalamus, hypothalamus, and brainstem nuclei (e.g., the periaqueductal gray; Damasio, 1995; Kringelbach, 2006).

Since the beginning of music making, composers have generated music expressing discrete emotions encountered in real-life situations, most typically happiness, sadness, and, to a lesser extent, fear, possibly by mimicking acoustic cues from affective vocalization, animal calls, or environmental sounds (Gosselin et al., 2005). Listeners, even those as young as 3-years-old, perceive and very quickly recognize discrete emotions in music (Dalla Bella et al., 2001; Juslin and Laukka, 2003). Furthermore, musical emotions in music from other cultures, like the ragas of Indian music, are recognized worldwide in a similar way (Balkwill and Thompson, 1999; Fritz et al., 2009; Thomson and Balkwill, 2010). Emotions perceived in music also activate brain areas, such as the amygdala, that are activated by emotions expressed in other modalities. Gosselin et al. (2005) obtained convincing evidence that in patients with medial temporal lobe resections encompassing the amygdala and in one patient with complete bilateral damage of the amygdala, the recognition of scary music was selectively impaired in the presence of intact perceptual skills of music such as the discrimination of music excerpts according to tempo, dissonance, or mode. FMRI evidence suggests that the amygdala, particularly the basolateral amygdala, is also activated by other musical emotions, such as unpleasantness and sadness (e.g., Koelsch et al., 2006; Mitterschiffthaler et al., 2007), confirming suppositions of its broader function in signaling danger, unpredictability, perceptual salience, or other phenomena sharing characteristics with fear. In music, however, discrete real-life emotions lose most of their threatening and aversive character due to the safe artistic context in which they are expressed.

Two fMRI studies have recently searched for the neural correlates of discrete emotions in music. First, Mitterschiffthaler et al. (2007) found that happiness felt in music (when contrasted with neutral music) increased activity in the limbic and paralimbic areas, namely striatal areas, parahippocampal gyrus, precuneus, medial, and superior frontal gyri, as well as the anterior cingulate cortex, whereas sadness felt in music (when contrasted with a neutral music) induced responses in the amygdala. However, direct comparisons between happy and sad music revealed only a few significant activations: happy versus sad music evoked activity in the left superior temporal gyrus (BA 22), and the reverse comparison did not reveal any significant activations. Brattico et al. (2011) found that perceived musical happiness contrasted with perceived musical sadness significantly activated a similar but larger region encompassing the secondary and associative auditory cortices (BA42/22) and extending to the insula (BA13). Sad contrasted with happy music elicited responses in the caudate and thalamus; similarly, activation of the thalamus has been consistently observed across fMRI studies of neural responses to sad faces (Fusar-Poli et al., 2009). Differences in stimuli and experimental paradigms may account for differences in findings between the two experiments: for example, Brattico et al. (2011) used participants’ self-selected instrumental music and songs (containing lyrics) from various genres, whereas Mitterschiffthaler et al. (2007) used well-known classical, instrumental pieces. However, a common finding of the studies taken together is that sad music does not strongly activate the brain when contrasted with happy music (Mitterschiffthaler et al., 2007; Brattico et al., 2011).

In line with evidence presented by theorists of the conceptual-act model that rebuts the concept of basic emotions as biologically hardwired, the pattern of physiological changes in the autonomic and central nervous systems associated with discrete emotions in music is not consistent (see, e.g., Krumhansl, 1997; Mitterschiffthaler et al., 2007; Brattico et al., 2011). In our proposal, we view discrete emotion recognition as a controlled process mediated by prefrontal and parietal brain structures and leading to the conscious reflection and categorical labeling of the bodily changes associated with a stimulus or an event. These bodily changes are governed by subcortical limbic and paralimbic structures and, when powerful enough, represent the process of the induction of discrete emotions from music. Hence, basic emotions are not supposed to be biologically hardwired as distinct physiological states but are rather mental phenomena that derive from changes in physiological states and a conscious mental process. In our chronometric proposal, the perception and induction of discrete emotions by music occurs after the early emotional responses and is reflected electrophysiologically by slow brain waves peaking at around 300–600 ms after the onset of the sound event, as preliminary evidence seems to indicate (Ellison et al., in preparation). Nothing is thus far known about the expression of emotions (from the sender), such as which brain structures and mechanisms are involved during a performance when a musician tries to convey an emotion.

### Aesthetic judgments

*Aesthetic judgment* (also sometimes termed appraisals) can be viewed as a special type of conscious evaluation typically directed at a human composition (like a musical piece or a painting) but also sometimes toward a natural object or event (a sunset, lightning, and so on). In musical and visual domains alike, the central component of such a judgment is the positive or negative outcome based on beauty or other criteria that the community considers relevant for the decision process (Jacobsen et al., 2004; Istók et al., 2009). Based on the results of a free-associations questionnaire, music-specific dimensions, such as melody, rhythm, harmony, and affective potential, are also important, particularly in musicians (Istók et al., 2009). Indeed, criteria for aesthetic judgments of music vary according to the style and the corresponding community of reference (e.g., von Appen, 2007). For example, criteria for aesthetic judgments of hip hop music are certainly divergent from those underlying the aesthetic appraisal of classical jazz music; to simplify, the former may rely on verbal complexity and the matching between word prosody and rhythm (e.g., Shusterman, 1991), whereas the latter may be judged based on the performer’s virtuosity, the mastering of jazz harmony rules, and the originality of improvisation (e.g., Gioia, 1988). The definition of these aesthetic criteria, including specific stylistic standards, is the aim of entire disciplines and is marginal to our purpose of unveiling the mental and neural chronometry of the aesthetic experience of music. Nevertheless, for our aims, it is important to notice that each listener has implicitly or explicitly internalized the rules and conventions of the musical style with which she is most familiar. In the words of Gallese and Freedberg (2007), “such processes might be precognitive and not always dependent on perception informed by cognition and cultural stock (as in much traditional aesthetics)” (p. 411). Taking this into account in our model, aesthetic judgment mainly follows cognitive processing of style-specific standards, as indicated by our psychophysiological findings (Müller et al., 2010; see Figure 1). The process of judging music according to certain criteria, an activity common to all listeners that sometimes only happens implicitly, is highly dependent on intentionality as well as external and internal contexts as we will illustrate later in this paper.

Aesthetic judgments, early emotional responses, and discrete emotions of music may be intertwined and hard to separate in a linear chronological sequence at the neural level. A very recent EEG and behavioral study purposely investigated one kind of aesthetic judgment of music, namely the attribution of positive or negative value according to the beauty dimension (Müller et al., 2010) and how it interacts with cognitive processing and emotional responses to sounds. After listening to 5 s chord sequences in which the last chord was manipulated in compliance with the rules of Western harmony, subjects were prompted by a visual cue to answer either the question “Is it beautiful?” or “Is it correct?” A late positive potential (LPP), lasting from 600 to 1200 ms after the last manipulated chord, was observed during both judgments but was larger when subjects judged beauty than when they judged correctness. In previous literature, the LPP has been associated with motivated, valenced attention to visual faces (Hajcak et al., 2006), erotic pictures (Briggs and Martin, 2009), words, and abstract black and white shapes (Jacobsen and Höfel, 2003). The larger LPP to beauty versus correctness chord judgments obtained by Müller et al. (2010) thus indicates an affective, motivational component in the computation of beauty judgments for chords. Furthermore, in a study of Japanese subjects using PET to measure brain metabolic activity, Suzuki et al. (2008) found that part of the dopaminergic reward system, namely the dorsolateral midbrain regions, was activated by listening to and rating the beauty of consonant chords irrespective of their major or minor keys (hence irrespective of their sad or happy emotional qualities) when contrasted with rating ugly dissonant major or minor chords. This finding was stronger with minor consonant chords, whereas the beauty ratings (contrasted with ugly ratings) of major consonant chords correlated with activity in the middle temporal gyrus. The authors interpret this result *post hoc* by associating minor consonant chords with additional pleasurable feelings in Japanese listeners due to a cultural preference bias for minor music. The issue is, nevertheless, still open since the neural correlates of the aesthetic chord judgments were not studied separately from those of the affective responses to them. In the subsequent sections of this paper, we capitalize on neuroimaging findings obtained with more complex musical sequences to propose separating the two processes of aesthetic emotions and judgments both in time, i.e., occurring in a specific temporal order, and in space, i.e., activating distinct neural systems.

What seems to distinguish aesthetic judgments of musical beauty from those in other domains (like in visual arts or literature) is the triggering of motion in the listener (e.g., Patel, 2008). It is a common observation that when we find a musical piece interesting or beautiful, we are motorically entrained, tap along with the beat, change our facial expressions, and (when possible) start to dance, sing, or play along. A musical experience, whether it consists of listening, performing, or dancing, is hence conceptualized as encompassing the whole body. This broad conception of music falls within the modern philosophical and neurobiological framework of embodied cognition (Lakoff and Johnson, 1980; Varela et al., 1992; Damasio, 1995), which posits that the human presence in the world and its cognitive understanding are mediated by the body and by the mutual interaction between different bodies and cognitive entities. Hence, applying the concept of embodied cognition to musical activities, some have proposed that the transfer of physical sound energy to the mental representation of music is embodied and requires motor and somatosensory body engagement (Molnar-Szakacs and Overy, 2006; Leman, 2007). An fMRI study by Kornysheva et al. (2010) provided empirical evidence for the engagement of premotor brain circuitry during aesthetic judgments. Eighteen subjects with little or no musical education were asked to give beauty or tempo judgments of slow to fast rhythmic patterns differing in beat subdivisions and played alternatively by wooden drums or metal drums. The contrasts between rhythms judged as beautiful and those judged as non-beautiful showed activation of the ventral premotor cortex and the cerebellum. In our framework, induction of motor activity and other physiological and bodily changes accompanies the flow of aesthetic processes (see Figure 1).

Interestingly, prefrontal areas like the superior frontal gyrus (BA 10) and the middle frontal gyrus (BA 9) of the orbitofrontal cortex coupled with the anterior cingulate cortex (BA 24) are recruited for beauty judgments of musical rhythms (Kornysheva et al., 2010). The explicit orientation of the subjects to process sounds aesthetically contrasted with instructions to focus on the tempo of the stimuli is sufficient to activate the orbitofrontal areas. Orbitofrontal cortex activation has previously been observed for situations that required the cognitive monitoring of events and sensory stimuli, which implicates this brain structure in aesthetic contemplation. It is important to note that the same regions of the brain, in particular the anterior orbitofrontal cortex (BA 10) coupled with the anterior cingulate cortex (BA 24), are also active during ratings of preference or beauty of faces, paintings, or geometrical shapes, and even during contemplation of paintings (Jacobsen et al., 2006; Kim et al., 2007; Cupchik et al., 2009). It has been suggested that these brain regions mediate cross-modal integration between subjective hedonic experience, visceral or bodily sensations, and evaluative judgment (Kringelbach, 2005).

The significance of the orbitofrontal cortex in aesthetic judgment has received support from other neuroimaging studies. A recent pivotal fMRI experiment by Ishizu and Zeki (2011) showed that a very small region of the medial orbitofrontal cortex, the A1 field, is activated by beautiful musical pieces and paintings (contrasted with ugly ones). A linear relationship between activation of A1 was even found with the intensity of the beauty experience. These findings led the authors to propose that the *aesthetic judgment* of beauty is hardwired in a specific brain area of the frontal lobe: anything activating that brain area would be experienced as beautiful. Such results confirm the need to broaden the classical comparison made between music and language (see, e.g., Peretz and Zatorre, 2003; Patel, 2008) to include other aesthetic domains like the visual arts, dance, and literature.

Two recent meta-analyses have identified several areas consistently involved in aesthetic appraisal and other aesthetically positive experiences. Brown et al. (2011) utilized a voxel-based meta-analysis of 93 imaging studies to identify brain regions activated by positive aesthetic appraisals across four sensory modalities. Areas including the supplementary motor area, dorsomedial thalamus, anterior insula, medial orbitofrontal cortex, and midbrain were active for positively judged auditory stimuli. An area in the right anterior insula was common to all sensory modalities (auditory, gustatory, olfactory, and visual). Although they did not find evidence of activity in the insula, Kuhn and Gallinat (2012) examined common areas activated by subjective positive judgments, including attractiveness, liking, or beauty, across 39 studies, and found regions of the ventromedial frontal lobe (including the orbitofrontal cortex), the anterior cingulate cortex, the left ventral striatum, the right cerebellum, and the left thalamus. While the identification of these areas as part of an aesthetic cross-modal circuit represents an advancement of the neuroesthetics field of research, the discrepancies between the two meta-analyses likely derive from the inclusion of a number of different types of aesthetic processes and modalities, thus highlighting the need for their analytic determination.

### Aesthetic emotions

*Aesthetic emotions*, such as awe, being moved, enjoyment, nostalgia, and chills or frissons, are, according to some scholars, the true emotions that can be induced (not simply expressed or perceived) by music (e.g., Konecni, 2008). The definition of aesthetic emotions, though, is still under debate. For instance, while not mentioning them explicitly, Koelsch (2010) argues for the legitimacy of musical emotions, as opposed to the artificiality of aesthetic ones, because they are controlled by the same brain structures associated with everyday emotions triggered by life events. Similarly, Juslin et al. (2010) oppose the use of the concept of aesthetic emotions when it is merely associated with any emotion evoked by a piece of art or when it represents refined emotions lacking goal relevance and action drive. As already briefly mentioned by Suzuki et al. (2008), aesthetic emotions should be regarded as distinct from other discrete emotions, such as sadness and happiness, as well as from aesthetic judgments. In the literature, they have been identified as emotions triggered by a work of art, i.e., in a context devoid of any obvious material effect on the individual’s wellbeing. In that sense, they have been contrasted with utilitarian or everyday emotions, which involve appraisal of the situation in relation to the individual’s goal and action oriented coping (Zentner and Eerola, 2010). In our recent work (Brattico and Pearce, 2013), we offer a compromise. Drawing on Sloboda’s (2010) distinction of music in everyday life versus in an aesthetic context, we suggest that casual (often inattentive) listening to music in everyday situations mainly induces basic emotions. Conversely, when a piece of music is listened to within an aesthetic context or performed with an aesthetic attitude (such as in a concert hall), special kinds of emotion might be generated, such as enjoyment, awe, and nostalgia. These can be considered truly aesthetic emotions.

We also propose that, as opposed to discrete emotions, which can be perceived and induced quickly after a very brief musical excerpt, aesthetic emotions are slow and often require listening to the piece of music as a whole. Their processing hence follows feature analysis, early emotional reactions, cognitive processing of musical rules, and discrete emotions (see Figure 1). In line with this, retrospective post-performance ratings could be considered as optimal for measuring experienced aesthetic emotions: they allow for the recollection of the entirety of an aesthetic event (although this would be biased toward the peak and ending experiences) and an assessment of its expressivity, and thus of its ability to induce discrete emotions (e.g., Juslin and Laukka, 2004; Laukka, 2007; Zentner et al., 2008). Zentner et al. (2008) asked over 800 attendees of a summer music festival in Genève to rate the appropriateness of a list of 66 adjectives in describing the emotions experienced during a performance. Very interestingly, according to confirmatory factor analyses, the 9-factor domain-specific model that best fit the listeners’ ratings included emotions that have been often described as aesthetic: wonder, nostalgia, transcendence, tenderness, peacefulness, power, joyful activation, tension, and sadness. Most of these emotions were positive, and even the sadness factor did not include aversive aspects typical of its utilitarian counterpart, such as feelings of gloominess or depression. The authors (Zentner et al., 2008; Zentner and Eerola, 2010) directly compared the 9-factor model to the basic emotion model, ascribing the differences between them to the specific properties of music. According to our proposal, time is crucial in accounting for the differences, as we will illustrate below.

Following Leder et al. (2004), we propose that aesthetic emotions and aesthetic judgments are the two outcomes of aesthetic processing. We further suggest that aesthetic emotions, when they are successfully triggered by music, succeed feature analysis, early emotional responses, and particularly core “liking,” cognitive processing, and discrete emotions in this temporal order (see Figure 1). In particular, discrete emotions in music are quickly perceived, induced (when possible), and assessed to determine the musical expressivity of a performance, which, in turn, might affect the induction of aesthetic emotions. Such a prediction stems from the currently sparse literature and calls for targeted empirical testing. In sum, we agree with Juslin et al. (2010) that an aesthetic emotion does not necessarily accompany an aesthetic judgment (there termed “response”) and that it has to be distinguished from conscious liking or preference (see below). In music, discrete and aesthetic emotions seem to be of central importance since aesthetic judgment is not necessarily explicitly present, such as in the common situation of incidental listening. Aesthetic emotions have been repeatedly indicated to be one of the primary reasons for wanting to attentively listen to music (Juslin and Laukka, 2004; Laukka, 2007; McDonald and Stewart, 2008), and even for choosing music as a profession (Sloboda, 1992).

An important type of aesthetic emotion is enjoyment. Similar to humor, music experience might be characterized by cognitive and affective elements (e.g., Moran et al., 2004). In humor, the cognitive element refers to understanding the disparity between the punch line and previous experience, whereas in music it might consist of detecting the violation of expected events (e.g., Huron and Margulis, 2010; Vuust and Kringelbach, 2010). The affective element may consist of the enjoyment derived from understanding the joke or the music. During this enjoyment moment, both in humor and in music, the perceiver experiences visceral and emotional reactions. FMRI and PET studies have demonstrated that musical pleasure recruits neural networks involved in the experience of reward and pleasure, including the ventral striatum (particularly, the caudate nucleus and the nucleus accumbens) and the orbitofrontal cortex (Blood and Zatorre, 2001; Koelsch et al., 2006; Salimpoor et al., 2011). These brain structures are active even when subjects passively listen to enjoyable music (as resulting from post-scanning tests) without being required to rate its pleasantness (Brown et al., 2004). Indeed, in depressed people, who have a decreased capacity for pleasure and enjoyment, favorite music compared to neutral music elicits significantly less activation of the ventral striatum than in healthy people, as evidenced by fMRI measurements (Osuch et al., 2009).

In music, a very strong aesthetic emotion of enjoyment in a listener or performer can sometimes be accompanied by certain bodily changes, such as chills, or goose bumps. We only briefly touch upon chills here, but extensive research has investigated this phenomenon (for a recent review, see Huron and Margulis, 2010). Although rare in occurrence (Huron, 2006; Juslin et al., 2010), these physiological responses represent an important bodily marker of emotional peaks (Grewe et al., 2009) and subjective enjoyment of music (Salimpoor et al., 2009). The neural correlates of chills during music listening have been discovered by way of PET and fMRI (Blood and Zatorre, 2001; Salimpoor et al., 2011). The intensity of chills, as measured by polygraph (e.g., heart rate, breathing, skin conductance, body temperature, blood volume pulse amplitude) and subjective ratings of pleasure, were correlated with activity in a broad network of brain regions including the ventral striatum, orbitofrontal cortex (BA 14), insula, anterior cingulate, cerebellum, supplementary motor area, and dorsal midbrain (possibly the periacqueductal gray), whereas it was negatively correlated with activation in the hippocampus, amygdala, cuneus, precuneus, and medial prefrontal cortex (BA 10 and 32). The subcortical regions associated with chills, such as the ventral striatum and periacqueductal gray, are also linked with pleasure in other mammals (Panksepp, 2009–2010). In a study in which the time course of the brain activity was investigated, the peak intensity of chills was positively correlated with dopamine release in the nucleus accumbens; on the other hand, anticipation, or the time immediately preceding peak pleasure, was correlated with activity in the caudate nucleus (Salimpoor et al., 2011). This highlights the importance of expectancy and anticipation in an emotional experience of music, as also emphasized by Vuust and Kringelbach (2010). Chills can be considered a subjective response, being highly variable between individuals, but some sensory features have been proven to relate with the chill response, such as high-pitched sustained crescendos similar to those characterizing the separation calls of neonates, sudden changes in harmony, and other musical events disrupting the expectations for incoming sounds based on previous musical knowledge. Thus, within our framework, chills are considered to be a physiological response at the interface between the automatic hardwired responses to sensory features of core “liking” and the subjective processes of the aesthetic emotion of enjoyment (see Figure 1).

The aesthetic emotion of nostalgia (for empirical studies of the emotional aspects of nostalgic experiences, see Wildschut et al., 2006; Janata, 2009; Trost et al., 2012) induced during music listening has also received the recent attention of neuroscientists and music psychologists. Indeed, the memory associations with life events that happened during a music listening experience dictate a strong emotional, both experiential and physiological, response to music (Juslin and Västfjäll, 2008). According to Konecni (2008), the aesthetic experience of music is determined in large part by episodic memory, with nostalgia considered the most important, and perhaps the only, emotion truly induced by music. Elicitation of memories and nostalgia are listed among the main reasons for listening to music and for the strongest bodily changes in both elderly and young adults (Laukka, 2007; McDonald and Stewart, 2008). The neural correlates of specific autobiographical memories associated with a musical piece have recently been investigated by Janata (2009) with a naturalistic paradigm. During fMRI scanning, subjects listened to 30 s excerpts of 30 pop and R&B songs (also containing lyrics) dating from their extended childhood (341 unique song excerpts across subjects) and rated them according to affective and autobiographical association scales. After fMRI scanning, subjects identified those songs that were judged as autobiographically salient and rated the strength of the associated emotional memories. The left dorsal medial prefrontal cortex (BA 8/9) reacted to the degree of autobiographical salience of the songs, likely establishing an association between structural tonality aspects and retrieval cues. Listening to autobiographically salient songs recruited both the left ventrolateral prefrontal cortex (particularly BA 44/45), also activated by structural violations of music (Tillmann et al., 2003; Koelsch et al., 2005), and the posterior cingulate gyrus, associated with other autobiographical memory tasks involving effortful retrieval demands. Hence, the findings demonstrate the power of music to evoke vivid memories and nostalgia. In doing so, music activates in a natural, spontaneous way the frontal network previously associated with effortful tasks in which subjects were required to retrieve episodes cued by single words or images (e.g., Svoboda et al., 2006). Further studies are needed, though however, to relate these activation patterns to the role of lyrics or melodies in nostalgia and autobiographical memories of music. For instance, using O^15^ PET to compare episodic versus semantic activations by familiar and unfamiliar melodic tunes, Platel et al. (2003) obtained similar but right-sided activations of the superior (BA 11) and medial (BA 8/9) frontal gyri along with the precuneus (BA 7); follow-up studies may elucidate whether this lateralization difference might be ascribed to the use of only melodic stimuli in Platel et al. (2003) as opposed to vocal music in Janata (2009).

Silvia (2005) has proposed the appraisal theory of emotion to account specifically for aesthetic emotions. This theory posits that a specific emotion stems from the adaptive outcomes of the evaluation or appraisal of an event in relation to a personal goal (Ellsworth and Scherer, 2003). For example, if the process results in the appraisal of an event as obstructive to personal goals, then an action tendency will result from high sympathetic nervous system arousal (Scherer et al., 2003); in contrast, if the appraisal is that it will be easy to cope with a situation, then the event does not control the emotion system and the individual can establish a new equilibrium (Ellsworth and Scherer, 2003). A first automatic appraisal, related to the assessment of whether a situation could be potentially dangerous for wellbeing, happens through a fast subcortical route similar to a reflex (Niedenthal et al., 2006). During fast appraisal the sensory valence and arousal of sound stimuli are processed, whereas a slow, cognitive appraisal relies on cortical processes for the evaluation of the individual’s capacity to cope with a situation (Niedenthal et al., 2006). According to Silvia (2005), a positive aesthetic emotion and judgment derives from the subjective appraisal of events according to the fulfillment of personal goals. For instance, interest, an aesthetic emotion deriving from the appraisal of novelty or complexity, combines with coping potential for the subjective feeling of being able to understand something that is new and complicated. However, it is known that the subjective conscious appraisal of discrete emotional states in faces is mediated by medial prefrontal cortex activation (e.g., Rubino et al., 2007). In the context of his Imagination, Tension, Prediction, Reaction, and Appraisal theory (ITPRA), Huron (2006) (see also Huron and Margulis, 2010) also indicates appraisal as an important affective mechanism in an aesthetic context independent of goal attainment: the immediate early emotional reactions that are caused, e.g., by a loud or dissonant chord (the latter resonating with the acoustic characteristics of screams or distress calls), are appraised in the musical context, which is harmless to the listener. Hence, an initial automatic negative reaction to a sad piece would be reframed within the aesthetic context and would hence produce the positive joyful feeling of aesthetic enjoyment. Similar to what we have conceived regarding aesthetic emotions, such an appraisal process is slower than the initial affective reactions to sounds and could be either conscious or below the level of awareness.

In sum, aesthetic emotions in our proposal succeed and integrate earlier affective processes, such as core “liking,” arousal, and other early emotional reactions, as well as perception, induction, and recognition of discrete emotions, leading to a (supposedly) longer-lasting emotional and bodily reaction. In line with Konecni (2008), we suggest as a working hypothesis that the longer timeframe of aesthetic emotions may sometimes be equal to that of mood induction processes.

### Conscious liking

Here we propose to distinguish between the early emotional reaction of core “liking,” discrete emotions, aesthetic judgments, aesthetic emotions, and conscious liking (see Figure 1). In contrast to enjoyment, liking includes a decisional, evaluative aspect. Most likely, conscious liking occurs in succession to–or even independently of–aesthetic judgments and emotional processes associated with listening to or performing music. Conscious liking (or disliking) has been conceptualized as a “long-lasting affective state” (Juslin et al., 2010) encompassing a general evaluation of an event on the basis of objective and subjective factors sometimes associated with positive (or negative) emotions. It has to be noted, nevertheless, that the act of evaluating music may itself affect the aesthetic emotion perceived and felt by the listener or performer. The term preference is often used as a synonym for liking, even though it emphasizes the static long-term aspect of the liking process. Aesthetic judgment, instead, relates to the outcome of a specific judgment along predefined aesthetic dimensions, such as beauty or formal structure, with the focus diverted from affective processes. As correctly indicated by Juslin et al. (2010), liking one piece over another does not necessarily involve the aesthetic evaluation of the piece’s quality as an art object but could be based on other individual factors, such as, we suggest, the assessment of the early emotional reactions and discrete emotions perceived and induced by the piece, the appraisal of its success in reaching a specific goal (like mood regulation), or even the conformity of the piece with the social codes and standards of a group of peers.

The comparison between the results of two distinct studies utilizing separate groups of subjects confirms the temporal distinction between aesthetic (beauty) judgments and liking judgments, and further indicates that liking judgments are slower in the mental chronometry than beauty judgments of the same musical material (five-chord cadences manipulated in their congruity with Western tonal harmony) when both judgment processes were contrasted to the process of rating correctness (Brattico et al., 2010; Müller et al., 2010). In the study by Brattico et al. (2010), the processing of liking judgments (in non-musicians), which were compared with correctness judgments of the music, corresponded to LPP electrophysiological responses peaking at around 1200 ms, and to reaction times of 453 ms ± 70 SD (from the end of the manipulated chord). In contrast, in the study by Müller et al. (2010), beauty judgments by non-musicians elicited long-lasting LPP brain responses, again distinct from the correctness judgments, beginning at 600 ms lasting up to 1200 ms. Reaction times for beauty judgments were 223 ms ± 31 SD (personal communication). Hence, due to the slower reaction times of liking judgments in the study by Brattico et al. (2010) in comparison to beauty judgments in the study by Müller et al. (2010), we have placed aesthetic judgments earlier in the chronometry of Figure 1 than conscious liking and preference. However, it is important to note that modulatory factors such as expertise (to be discussed later) might invert this temporal order. This hypothesis needs to be tested empirically.

An innovative study by Altenmüller et al. (2002) examined the neurophysiological correlates of musical liking by EEG recordings obtained while subjects listened to and rated 120 15 s musical excerpts from classical, pop, and jazz genres as well as environmental sounds. The EEG analysis focused on lateralization effects between left and right pairs of scalp electrodes and found that liking elicited larger brain oscillations in the left frontotemporal scalp regions as compared to the right, whereas brain oscillations to disliking were lateralized more to the right anterior brain regions. Neutral music generated bilateral brain activity, further confirming the modulation of music processing by affect. Interestingly, these effects were more pronounced in females than males. A second, more recent study (Flores-Gutiérrez et al., 2007) combined EEG (with 16 subjects) and fMRI (with 6 subjects) to study the neural processing of 10 min of music by the classical composers Bach and Mahler (presented in blocks of 30 s), mainly liked by subjects, and 10 min of music by the contemporary composer Prodromidés, overall disliked by subjects. As in the study by Altenmüller et al. (2002), liked music activated left-hemispheric brain regions, and in particular the auditory cortices (BA 41 and 42), the middle temporal gyrus (BA39), and the cuneus, whereas disliked music generated brain responses in the bilateral inferior frontal gyrus and insula (along with left-hemispheric activation of the middle frontal gyrus). To note, in both of these studies, the authors did not refer to liking as a definite psychological phenomenon but rather referred to positive or negative emotions or valence category, thus emphasizing the need for conceptual clarification in the field. In a third fMRI study on social influence (Berns et al., 2010), liking was assessed by asking adolescent participants to give likability ratings of 15 s excerpts from 20 pop songs taken from MySpace.com. Activations in the bilateral caudate nucleus, the bilateral supramarginal gyrus and the left cingulate cortex, as well as in several smaller clusters in frontal (somatomotor and associative) and temporal (auditory) regions were found to positively correlate with liking in adolescents. However, a direct comparison of liking processes in adolescents and adults, which would evidence the maturational course of these affective responses to music, has yet to be conducted. Furthermore, a direct comparison between spontaneous enjoyment from passive listening, pleasantness ratings, and judgments of conscious liking has to be attempted in order to determine the role of cognitive and associative areas in evaluative liking processes as compared with emotional pleasurable responses.

Familiarity with an object affects conscious liking according to an inverted U curve function. This so-called “mere exposure” phenomenon was first identified by Zajonc (1968) and seems to be valid also in the musical domain, as aesthetic judgments for music followed the inverted U curve with the highest ratings for medium exposure (Schellenberg et al., 2008). In general, familiarity with a set of musical rules determines preference. For instance, constant repeated exposure to a set of sounds (perhaps combined with innate predispositions) generates prototypes, or ideal exemplars of a perceptual category. In Western tonal music, prototypes have been identified in the 12 sounds of the chromatic scale and the major and minor triads, used as anchor points for the induction of tonal hierarchical pitch processing (Brattico et al., 2009). The aesthetic value of prototypes and familiarity with musical rules is demonstrated by the finding that 6- and 7-year-old children preferred diatonic over non-diatonic melodies and judged them to be more beautiful (Krumhansl and Keil, 1982; Nieminen et al., 2011, 2012). Indeed, in comparison to unfamiliar music, familiar music (liked or disliked) induced much stronger blood-oxygen-level-dependent (BOLD) activations of emotion-related limbic and reward areas, including the right nucleus accumbens, bilateral putamen, bilateral amygdala, right anterior cingulate cortex, left hippocampus, bilateral supplementary motor area, and left orbitofrontal cortex, thus suggesting that familiarity plays a role in the neural basis of musical enjoyment and conscious liking (Pereira et al., 2011).

The distinction between aesthetic judgment and conscious liking proposed here (see Figure 1) is to be taken with caution. Unpublished data from our labs evidence a correlation between ratings of beauty and liking of the same musical material extracted from symphonic movie soundtracks. We can, nevertheless, suppose that such correlations may vary depending on musical genre, and the age and musical expertise of the subjects.

### From genre preference to musical taste

As briefly mentioned above, preference can be conceptualized as the static outcome of an aesthetic process of evaluating a musical piece, and in this sense, it can be used as a synonym for conscious liking. However, preferences can be imagined to be stable over time, whereas liking is better viewed as an ongoing process. This is particularly true when considering preferences for a whole genre or style of music. Social factors are known to affect preferences for musical styles in young listeners. In particular, the preferences of the parents and siblings of children determine their own musical preferences (Roulston, 2006). Gender stereotypes also play a role in the choice of an instrument when starting to play in childhood (Harrison and O’Neill, 2000). Furthermore, music psychology literature has repeatedly suggested that musical preference is at least partially determined by the individual’s personality. For instance, in a study of approximately 3500 individuals, Rentfrow and Gosling (2003) found four music preference dimensions (Reflective and Complex, Intense and Rebellious, Upbeat and Conventional, and Energetic and Rhythmic), each of them differentially correlated to the personality dimensions of the Big Five questionnaire (Extraversion, Agreeableness, Conscientiousness, Emotional Stability, and Openness) as well as to self-views and cognitive ability. A recent questionnaire study on musical preferences, though, obtained only a weak correlation between personality traits and favorite musical genres and instead emphasized the relationship between gender and increasing age, on the one hand, and the complexity of music, on the other hand, with females showing higher liking for classical, mainstream, folk, alternative rock, Latino and Music Of Black Origin (MOBO), and men for jazz, dance, rock, and functional music (North, 2010).

The accumulation of musical preferences from individual expert musical knowledge, social attitudes, previous situations, and successful emotional regulation by music results in the distinctive musical *taste* characterizing an individual. In other words, musical taste is intended here as a long-term set of preferences, aesthetic judgments, values, and attitudes. These are affected by the listener’s characteristics, namely her biography, age, gender, personality, and social status. In a feedback loop, musical taste, together with the subject’s immediate context variables such as current mood, attention, arousal, and current social attitudes, determines conscious liking (see Figure 1). According to previous research, we could venture to say that the feedback loop between taste and liking is pliable in early adulthood as compared with childhood or later adulthood (LeBlanc et al., 1996; for a review, see Hargreaves and North, 2010). During the years of late adolescence and early adulthood, individuals are explorative and open to listening to various musical genres, consistent with the “open-earedness” hypothesis (LeBlanc et al., 1996; Hargreaves and North, 2010), which in turn leads to the formation of the musical taste that will become more crystallized in future years. Furthermore, in a pioneering study, adolescents changed their liking ratings to conform with their reference group when popularity was revealed (Berns et al., 2010), thus confirming that adolescents are more susceptible to the influence of their peer group in forming preference.

## Modulating Factors

### Internal context

We cannot neglect the importance of context for any aesthetic and affective experience of music. With the term context, we refer to all the external or situational as well as internal or individual variables that contribute to a musical experience. We have placed these factors in Figure 1 external to the chronometry as they likely play a role throughout the aesthetic experience, on a different time scale than the other processes. Hargreaves and North (2010) have pointed out the modulatory effects of both the listener (or the *internal context* in our terminology) and the situation (namely the external context) on musical preference and other aesthetic responses. With respect to internal context, the most studied variable is expertise in music and its long-term knowledge. Recent scientific efforts have examined both the effects of the interaction between music and mood, defined as the stable background affective state of an individual, on the act of choosing music as well as the effects that music has on changing the current mood. Among other aspects constituting an internal context of a listener or performer, we can list attention intentionality, and attitudes in addition to age and personality (which were already discussed in the section related to musical preferences).

#### Attitudes

An important modulator of the aesthetic experience of music, even before it commences, is attitude. This can be described as stored long-term memory evaluations, entailing a valence aspect, a knowledge component, and a behavioral tendency, which are activated automatically and allow us to frame everyday situations quickly (Petty et al., 1997). Research on the influence of attitudes has received some attention within the framework of empirical aesthetics of figurative arts (see Jacobsen, 2010). To our knowledge, so far only two studies have addressed the neural correlates of top-down interpretation in music (e.g., Steinbeis and Koelsch, 2009; Berns et al., 2010). In an fMRI study, Berns et al. (2010) investigated how the popularity ratings of a certain song by adolescents influence other adolescents’ liking of that song. Surprisingly, they observed that popularity ratings affected the brain activity of the bilateral anterior insula and the anterior cingulate, regions not corresponding to those that would be expected to relate to musical pleasure, namely the orbitofrontal cortex and the ventral striatum. Contrarily, the activity in the ventral striatum decreased when subjects changed their rating after knowing the song popularity, indicating a personal cost when changing the direction of a rating. Using the same musical stimuli for each condition, Steinbeis and Koelsch (2009) compared the brain areas activated when subjects believed that the music was composed by an artist to the brain areas activated when they believed that it was produced by a computer. This subtle experimental manipulation, leaving the acoustic content intact and altering only the top-down interpretation of the stimulus, was sufficient to activate brain structures such as the anterior medial frontal cortex, the superior temporal sulcus, and the temporal poles, which are responsible for social cognition and, in particular, for the attribution of a viewpoint to another person. Similar findings have been obtained in the context of visual arts by Cupchik et al. (2009): activation of a visual associative area (the right fusiform gyrus) was found when subjects observed a painting with a pragmatic attitude whereas activation of the bilateral insula and of the left lateral prefrontal cortex was obtained when subjects observed the same painting with an aesthetic attitude focused on beauty judgments. Any listener or performer approaches music with an attitude. When the attitude is emotionally tinged and includes preparatory processes for evaluating the beauty of a musical piece or the mastering of a musical performance, then an aesthetic attitude toward the music has been established, and an aesthetic experience can ensue over time. Aesthetic attitudes are further affected in a feedforward loop by internal contexts, such as expertise, attention, intentionality, and mood (see Figure 1).

#### Expertise

Expertise, whether acquired through formal training or resulting from informal musical activities (Folkestad, 2006), internally modulates the aesthetic experience of music by shaping memory systems at most levels of processing. In the literature, subjects with at least 5 years of musical training in conservatories or other music academies and/or who earn their living by performing music are typically classified as musicians or music experts. Recently, a questionnaire specifically assessing musical competence acquired from informal musical activities, along with that acquired from academic training, was developed (Müllensiefen et al., 2010); however, there is thus far little research on the subject of informal musical activities, despite their likely impact on the aesthetic experience of music. Here, we thus focus on formal musical training, which consists of (mainly) attentive, intensive practice encompassing several hours per day for many years and usually starting at an age earlier than 6 years (Altenmüller, 2009). First, this particular kind of training leads to the acquisition of motor skills, such as the fine and fast movements required to play an instrument. The neural consequences of the acquisition of these motor skills is the plastic shaping of the neural networks for somatosensory representation of the fingers involved in the playing movements (Elbert et al., 1994), the densification of the corpus callosum (the bundle of neuronal fibers connecting the two cerebral hemispheres; Schlaug et al., 1995), and the enlargement of the gray matter of both the primary motor cortex (Gaser and Schlaug, 2003) and the cerebellum (in male musicians only; Schlaug, 2001). Importantly, all of these changes are correlated with the age of onset of the musical training. In non-musicians who have learned to play a simple melody, the motor circuit encompassing “mirror” or “echo” neurons is activated when they listen to the learned familiar melodies but not when they listen to equally familiar but untrained melodies (Lahav et al., 2007). Indeed, the same auditory-motor regions in musicians, including the posterior superior temporal gyrus (planum temporale) and the motor areas, are active while listening to or while playing a familiar piece (Baumann et al., 2005; Bangert et al., 2006), indicating a higher coupling of these systems as a consequence of musical training (cf. Zatorre et al., 2007).

Second, musicians who received formal training–and even other kinds of music experts (e.g., individuals without performance skills or theoretical knowledge but possessing a rooted familiarity with certain genres through listening and taking part in musical subcultures)–acquire auditory perceptual skills. For instance, psychoacoustic research has showed that musicians exhibit a smaller discrimination limen (the acoustic parameter difference between two tones in a same-difference task) due to attentive listening and practice. Previous experience with musical sounds shapes the perceptual feature analysis of pitch contour in language already at the brainstem level (Wong et al., 2007). In the auditory cortex, the neural representation of isolated musical sounds, as reflected by the N1 electrophysiological response, is more efficient in musicians, particularly for those instrumental timbres that are most familiar (Pantev et al., 2001). Discriminating musical sounds based on their sensory memory traces is finer and faster in musicians: a slight mistuning of chords elicits MMN brain responses in musicians only (Koelsch et al., 1999; Brattico et al., 2009), and interval changes in sound patterns induce faster MMN responses in musicians (Brattico et al., 2001; Fujioka et al., 2004). The special type of exposure to music occurring in musicians also modulates the content of their long-term schematic memory for musical prototypes. Brattico et al. (2009) found that the neural responses in the auditory cortex to a change from a prototypical chord to a non-prototypical one were enhanced in musicians compared to non-musicians, and that their strength was also positively correlated with the length of musical training. This perceptual fine-tuning of auditory skills in musicians is most likely a consequence of the increased volume of gray matter layers in their bilateral primary auditory cortices: such structural changes in the brain accompanied by superior musical skills have been found in adults (Schneider et al., 2002) and, most importantly, in children after only 15 months of musical training (Hyde et al., 2009; for a recent review, see Kraus and Chandrasekaran, 2010).

Third, musicians are extensively trained in music theory and hence they attain explicit schematic knowledge and related cognitive skills to recognize and manipulate the properties of a musical system. For instance, musicians are able to name tones and chords by their musical labels, to segment musical phrases and compose new ones on the basis of a set of rules, and the like. The abstract contents of schematic memory for music are hence more accurate and more quickly activated in musicians, as behavioral and neural data demonstrate (Bigand and Poulin-Charronnat, 2006). Explicit knowledge of a set of rules valid for Western music, i.e., tonal harmony, defining how chords should succeed one another in tonal music (Piston, 1962), is reflected in an enlarged ERAN neural response to incongruous chords at the end of an authentic cadence in musicians as compared with non-musicians (Koelsch et al., 2002; Brattico et al., in press). Processes of restructuring the tonal context are also reinforced by long-term schematic knowledge of music, as demonstrated by increased P600 brain responses to incongruous notes or chords at the end of sequences (Besson et al., 1994; for reviews, see Koelsch and Siebel, 2005; Patel, 2008; Levitin and Tirovolas, 2009). Even long-term rhythmic knowledge in musicians is reflected in modified brain activity. For instance, coupling between premotor and auditory areas in musicians increases while perceiving different rhythms (Grahn and Rowe, 2009). Similarly, working memory brain areas (such as the right dorsolateral prefrontal cortex) are more active during the production of complex meters by musicians as compared with non-musicians, whereas motor areas are recruited similarly across musicians and non-musicians (Chen et al., 2008).

In sum, feature analysis, short-term memory, and the cognitive processing of music are demonstrated to be affected by expertise derived from intensive, decades-long musical training. In contrast, no data has been collected on the role of expertise in early emotional reactions, discrete emotions, or conscious music liking, except for very recent findings on augmented auditory neural responses and automatic nervous system reactions in musicians (as compared with non-musicians) to sensory dissonance (or core “liking”; Brattico et al., 2009; Dellacherie et al., 2011). The renewed attention toward emotions in the field of music neuroscience will probably fill in the current gap in the next few years.

#### Mood

The long-lasting and low-intensity affective state of an individual, i.e., the current *mood*, influences the decision to approach an aesthetic stimulus, the function of the stimulus for the individual, and her evaluation of liking the stimulus (for a recent review, see Konecni, 2010). For instance, in a forced-choice behavioral experiment (Breckler et al., 1985), listeners could select one out of five sonic alternatives every 15 s, which included complex or simple soothing music and complex or simple aversive sounds. Listeners often placed aversive sounds in the beginning of the experiment and the most-liked piece of music at the end of the experiment for final listening enjoyment. Interestingly, the second most-liked piece was used to intersperse and offset the negative mood induced by aversive sounds. Additionally, the internal context or mood of the individual approaching an aesthetic experience may render its outcome positive or negative: while moderately positive moods shift the individual into a creative, aesthetic attitude, extremely positive or negative moods reduce attention and are hence not optimal for the reception or production of an aesthetic event (Wilson and Gilbert, 2003). In turn, a musical aesthetic experience might by itself change the initial mood of an individual, who may even intentionally seek its changing potential. Indeed, a predominant reason for listening to music in everyday life is its ability to regulate mood (Sloboda and O’Neill, 2001; Laukka, 2007), and music is also the most frequent source of mood regulation in everyday life (Thayer et al., 1994), particularly for adolescents and music amateurs (Saarikallio and Erkkilä, 2007; Saarikallio et al., 2013). So far, the neural processes involved in mood regulation have been studied mainly in the visual domain and include the up-regulation or down-regulation of the amygdala, depending on the initial emotional state and the final mood regulation goal to be achieved. The brain structures sending signals to the amygdala to cognitively reframe an affective state are the prefrontal cortex and the anterior cingulate cortex (Ochsner and Gross, 2005). In particular, the right ventrolateral prefrontal region has been found to be more active during the down-regulation of negative emotions derived from aversive pictures (Wager et al., 2008). One fMRI study demonstrated that the responses to dissonant chords in limbic and associated structures (e.g., the left precuneus and right amygdala) are down-regulated when performing a cognitively demanding task (Pallesen et al., 2009). These studies illustrate the feasibility of studying the cognitive regulation of emotional experiences of and through music within an aesthetic framework.

#### Attention and intentionality

*Attention* modulates any kind of emotional experience: in an fMRI study, the expectation of a picture enhanced activity in the medial prefrontal cortex, amygdala, and dorsal midbrain for emotional but not neutral pictures (Bermpohl et al., 2006). It has to be noted that music often takes place in situations where listening is not the main activity (Sloboda and O’Neill, 2001). In the laboratory, when such incidental listening was reproduced by diverting attention with a timbre discrimination task, subjects liked the sad musical excerpts more and were less accurate in recognizing the happy excerpts (Schellenberg et al., 2008). Further, the U curve of preference after repeated exposure was reproduced during focused listening (the mere exposure effect), but it was replaced by a monotonically rising line in the incidental listening condition (Schellenberg et al., 2008). According to Jacobsen (2010), contemplation and distraction are major constituents of the aesthetic experience along with judgment, appreciation or enjoyment, and preference. Aesthetic distraction occurs when attention is involuntarily switched toward a beautiful entity or object and aesthetic processing commences. In contrast, aesthetic contemplation requires mental effort and reflection leading to a subjective evaluation though, unlike the aesthetic judgment, without an overt verdict or judgment over the contemplated object. As also demonstrated by an electrophysiological study in the visual modality (Höfel and Jacobsen, 2007), these processes are marked by different degrees of attentional control and cognitive involvement. Although the aesthetic enjoyment of music is a very common phenomenon and most likely, as previously mentioned, one main reason for our daily seeking of music, we cannot maintain that listening to (or performing) music always creates a full aesthetic experience. According to a study by Sloboda and O’Neill (2001) using the experience sampling method, about 44% of the events recorded involved music, but music was listened to intentionally and attentively in only 2% of them.

Related to this, *intentionality* also has a crucial role in our model. The subject is viewed as an active agent with expectations, goals, and predictions that govern her decision to consciously and attentively approach an aesthetic experience and influence what kind of music she chooses to listen to and how. When a listener or performer initiates an aesthetic musical event, she brings to mind a representation of it. If the event has been experienced many times before, the mental representation is supported by a prototypical event and becomes veridical. Social psychologists have used the term affective forecasting to refer to the implicit or explicit predictions formed by individuals about future feelings in response to approaching events (Wilson and Gilbert, 2003). For instance, when subjects were armed with expectations that a film would be funny, they looked at it for less time than when they did not have any expectation about it (Wilson and Gilbert, 2003). Liking a musical piece that would otherwise not be appreciated without positive expectations might take place as a self-fulfilling prophecy (Wilson and Gilbert, 2003). Indeed, the choice and preference for a particular piece of music can also be directly linked to the evaluation of its success in achieving arousal-based goals (Hargreaves and North, 2010). For instance, a relaxing low arousal piece of music is selected after a hard day at work, or a fast high-arousal piece is chosen for a gym class. The neural correlates of attentive processes during musical enjoyment and preference, which lead to the verdict of loving or hating a musical piece, are still to be investigated within the musical domain.

As indicated by the dashed lines in Figure 1, often only some processes constituting an aesthetic experience are activated; others remain dormant and are triggered only in the case of exceptionally beautiful or pleasurable events (as hypothesized for the visual domain by Jacobsen, 2010). When aesthetic processes are dormant due to distraction during incidental listening, discrete emotions are prioritized over “aesthetically tinged” ones (Sloboda, 2010, p. 503). In these cases, the aesthetic experience is incomplete and does not lead to the authentic aesthetic responses, which we have indicated here as consisting of aesthetic emotions, aesthetic judgments, and preference.

### External context

According to Leder et al. (2004), the same object is apprehended and evaluated differently when considered a piece of art. By itself, the external context might implicitly determine an aesthetic attitude oriented toward beauty judgment, aesthetic emotions, and liking in the listener or performer. The physical environment in which music is listened to or performed constitutes the most obvious *external context*. According to several studies, music in everyday life is listened to mainly while driving, socializing with friends, alone at home, exercising, or walking (for a review, see Sloboda, 2010). Instead, some architectonically beautiful places with fine acoustics, such as a Medieval cathedral, seem to be optimal for determining the efficacy and intensity of an aesthetic musical experience and for inducing aesthetic awe (Konecni, 2008), in line with the notion that music is a multi-systemic phenomenon involving the sensory, affective, cognitive, and motor systems. It is already known in the literature that emotional experiences of music, as measured by psychometric ratings and physiological and brain recordings, and congruent emotions, conveyed via the visual channel, enhance each other (Baumgartner et al., 2006; Eldar et al., 2007). Future investigations should aspire to test any reinforcement of the aesthetic experiences of music as defined here (involving emotions but also judgment, liking, body engagement, and cognition) by isolated versus multiple sensory systems. Also it is crucial to understand whether and how the manipulation of a (real or imagined) location for a music event would affect the brain responses to it.

Another relevant external context variable is the social environment, such as the presence of other listeners, performers or dancers, or in contrast, the solitude of a room. A recent study by Sutherland et al. (2009) aimed at empirically demonstrating the effect of social context on strong experiences or aesthetic emotions (to use our terminology) derived from music, as reflected by the induction of chills. However, contrary to expectations, there was no difference between the experimental conditions of listening alone and listening in a group. This study cannot be considered conclusive evidence since the social context of an aesthetic experience, such as the gathering of crowds for a concert, cannot be easily reproduced in a laboratory setting. An alternative approach adopted by, among others, Juslin et al. (2008) utilized the experience sampling method to investigate the emotional responses to music in everyday life. For several days, subjects carried a portable palmtop computer that emitted a sound signal to prompt them to fill out questionnaires about their current experiences. The frequency of musical emotions experienced by subjects was modulated by a social or solitary environment, with happiness-elation, pleasure-enjoyment, and anger-irritation being most common when listening to music with others, and calm-contentment, nostalgia-longing, and sadness-melancholy occurring more frequently when listening alone. Recent technical advances have rendered psychophysiological research with experience sampling methodology feasible, which could help us understand the influence of physical environment on a musical aesthetic experience.

The social environment represented by peers may also profoundly determine not only consumption decisions and listening choices (by acting on musical preferences), but also the actual emotional responses to a musical piece. A recent study by Egermann et al. (2009) showed that those subjects who knew beforehand the ratings of arousal and valence by preceding participants were influenced in their emotional recognition as opposed to the ratings of subjects not receiving any feedback. These results can be explained by the phenomenon of compliance, described by Hargreaves and North (2010; p. 531) as the “desire to conform with the opinions of valued social groups, so as to confer increased status within those groups.” One could hence assume that social feedback would markedly determine the aesthetic reactions of listeners, possibly to a larger extent when subjects are of a particular age, such as adolescence, as revealed by the previously described fMRI study by Berns et al. (2010).

## Conclusion and Implications

Here we have delineated a mental and neural chronometry of the aesthetic processes involved in music listening and/or production, beginning with lower-level pre-attentive feature analysis, followed by discrete and aesthetic emotional processing, and proceeding to conscious liking and top-down aesthetic judgments. We also cover the contextual modulatory factors operating on different time scales and interacting (possibly non-linearly) with each chronological stage of the aesthetic experience. This framework of temporal successions of processing stages is to be considered an initial proposal for reframing scientific music research within neuroesthetic studies of other art experiences (for another recent effort in this direction, see Brattico and Pearce, 2013). The research that inspired the current model is still sparse but represents an encouraging starting point for further endeavors. By laying out the different sequential processes and factors contributing to an experience of music, particularly with a focus on the aesthetic dimension, we provide a novel contribution to the currently available theories on music processing (cf. Peretz and Zatorre, 2003; Koelsch, 2011), which have thus far largely neglected the top-down affective processes, attitudes, and contexts typical of an aesthetic situation and instead have mainly focused on lower or intermediate emotional and perceptual levels. Similar to our proposal, influential frameworks dedicated to the visual arts (but completely neglecting music) adopted an information processing viewpoint, in which the aesthetic experience is decomposed into an ordered series of stages on the basis of psychological or neuropsychological evidence (Chatterjee, 2003; Leder et al., 2004; Jacobsen, 2006). Nadal et al. (2008) put forward a model for visual aesthetics attempting to integrate neuroimaging evidence from three studies into the neuropsychologically grounded model by Chatterjee (2003). The novelty of our contribution relies on focusing on neuroscience findings as a foundation for a temporally and spatially ordered framework of the aesthetic experience of music. Figure 1 highlights the possible neural substrates of each psychological subcomponent of the musical aesthetic experience. As compared with the model by Leder et al. (2004), we propose preference as the third important outcome of an aesthetic experience, along with aesthetic emotions and aesthetic judgments, and we propose novel hypotheses for research, such as the importance of differentiating between mental and neural mechanisms underlying discrete and aesthetic emotions.

Indeed, the field of neuroesthetics of music, which could be considered an umbrella for the research agenda put forth here, is still in its infancy; the first review dedicated to it was written only very recently (Brattico and Pearce, 2013). As Chatterjee (2011) wrote in reference to the neuroesthetics of visual art, “with a field so young, development in any direction would be an advance” (p. 58). We here attempted to provide a novel context for the neurosciences of music in which processes such as the cognitive understanding of melody, rhythm and harmony, chills, preference, and decision-making, previously studied as distinct mental domains, can be integrated into the unique experience of aesthetic appreciation. From this framework, neuroesthetics hypotheses concerning music could be tested in future.

Nevertheless, we should ask ourselves what an aesthetic stance has to offer to the advancement of research in music neuroscience. Within our chronometry, two outcomes of the aesthetic experience of music have thus far been widely neglected by music neuroscientists, namely beauty judgments and liking or preference. The first neglected area of research, which we hope will receive a stimulus from the delineation of the current framework, relates to beauty in music. The judgment of beauty and the hedonic response associated to it, namely enjoyment, represent a pivotal motivation for our music seeking behavior. Evolutionarily, beauty, being a cue for health and vigor, has a role in human mate selection and the production and appreciation of beautiful objects or sounds has functioned in rituals for enhancing social cohesion (Brattico et al., 2009/2010). Conceptualizing music behavior from the perspective of beauty and aesthetic enjoyment rather than from its cognitive benefits (such as the cross-domain transfer of music abilities to other executive and language functions; see Schellenberg and Winner, 2011) could help in understanding the efficacy of music, especially when liked and preferred (see Garza Villarreal et al., 2012), on pain reduction, on the emotional and cognitive states of brain-lesioned patients, for depressed and schizophrenic patients, and on several other clinical conditions (for reviews, see Lin et al., 2011; MacDonald et al., 2012; Särkämö and Soto, 2012). Indeed, aesthetic evaluative processes, such as liking and beauty judgments, while being the least studied by neuroscientists, are the most common ones in everyday musical behavior. The reasons for purchasing the songs of one artist versus another are of interest for the music industry and the related marketing research. Discovering the neural bases for these choices might have direct applications without, we believe, impoverishing the variety of creative production, since preliminary research indicates a wide range of preferences based on numerous individual factors (Rentfrow and Gosling, 2003; North, 2010).

We also hope that another impact of our proposal would be the shift of attention from pleasurable aesthetic emotions to other aesthetic ones, such as provocation, awe, surprise, and nostalgia. Such a paradigmatic shift might enable us to understand the nature of positive aesthetic emotion, which goes beyond sensory pleasure and likely derives from the degree of knowledge or cognitive mastering of the relevant musical style (as proposed by Leder et al., 2004). Ultimately, the delineation of the temporal order, neural substrates, and psychological mechanisms composing each single process during a musical aesthetic experience will shed light on the nature of the experience itself, contributing not only to understanding a brain function but also a typically human behavior, namely music.

## Conflict of Interest Statement

The authors declare that the research was conducted in the absence of any commercial or financial relationships that could be construed as a potential conflict of interest.
